# Half Empty *and* Half Full? Biased Perceptions of Compassionate Love and Effects of Dyadic Complementarity

**DOI:** 10.1177/01461672231171986

**Published:** 2023-05-26

**Authors:** James J. Kim, Harry T. Reis, Michael R. Maniaci, Samantha Joel

**Affiliations:** 1 Western University, London, Ontario, Canada; 2 University of Rochester, NY, USA; 3 Florida Atlantic University, Boca Raton, USA

**Keywords:** partner perceptions, accuracy and bias, DRSA, love, complementarity, dyadic

## Abstract

The prevailing theory on relationship judgments for interaction attributes suggests individuals tend to underestimate a romantic partner’s expressions of compassionate love and that such underestimation is beneficial for the relationship. Yet, limited research has incorporated dyadic perspectives to assess how biased perceptions are associated with both partners’ outcomes. In two daily studies of couples, we used distinct analytical approaches (Truth and Bias Model; Dyadic Response Surface Analysis) to inform perspectives on how biased perceptions are interrelated and predict relationship satisfaction. Consistent with prior research, people demonstrated an underestimation bias. However, there were differential effects of biased perceptions for actors versus partners: Underestimation predicted lower actor satisfaction but generally higher satisfaction for partners. Furthermore, we find evidence for *complementarity* effects: partners’ directional biases were inversely related, and couples were more satisfied when partners had opposing patterns of directional bias. Findings help integrate theoretical perspectives on the adaptive role of biased relationship perceptions.

Romantic relationships are centrally characterized by the everyday positive relationship behaviors partners enact to demonstrate their care, validation, and understanding for one another (Reis & Shaver, 1988). A robust body of research has examined the relational benefits associated with various positive relationship behaviors, such as love, responsiveness, and accommodation, demonstrating their crucial importance for optimal relationship functioning (e.g., Lewandowski et al., 2014). At the same time, research indicates that the impact of positive relationship behaviors can crucially depend on the way in which they are perceived. Indeed, significant research efforts have been directed toward identifying whether individuals tend to be accurate or biased in their perceptions of romantic partners, and how specific patterns of perception are associated with relationship outcomes (e.g., Gable et al., 2003; Lemay et al., 2007; Reis et al., 2004).

In the current research, we examined daily expressions and perceptions of compassionate love behaviors, given their relevance for addressing key questions surrounding partner judgments that focus on the interdependence between partners (i.e., *interaction attributes*; Fletcher & Kerr, 2010). To date, there remains a lack of empirical consensus on the dyadic patterns underlying partner perceptions, and whether it is better for romantic partners to view the “glass as half-full or half-empty” in their relationship judgments. Here, we sought to examine: (a) how positively or negatively biased perceptions are linked with *both* relationship partners’ outcomes, (b) whether partners tend to be biased in similar ways, and (c) whether relationship outcomes differ if two partners exhibit a similar or different pattern of bias. We used two contemporary models of interpersonal accuracy (the Truth & Bias Model and Dyadic Response Surface Analysis) to answer whether it is better for partners to identify patterns of accuracy and bias for compassionate love behaviors and shed light on their dyadic associations with relationship satisfaction.

## Accurate and Biased Perceptions in Relationships

Research has long been interested in how romantic partners’ evaluations and judgments are often characterized by bias, with prevailing theories suggesting that biased perceptions may serve adaptive functions for relationship satisfaction and stability (e.g., Fletcher & Kerr, 2010; Haselton & Buss, 2000). Yet, findings on this topic have not always appeared consistent. For example, the literature on *positive illusions* suggests romantic partners tend to wear “rose-colored glasses” insofar as individuals tend to idealize their partners’ qualities and behavior or hold overly optimistic views about their relationship. Generally, this body of work suggests that such a bias (“seeing the glass as half-full”) may be beneficial for relationships as individuals who see their partners in a more favorable light than is perhaps warranted tend to report more favorable relationship outcomes, including higher levels of satisfaction and commitment, (e.g., Miller et al., 2006; Murray et al., 1996). At the same time, research additionally provides support for the self-verification theory, which holds that people tend to want partners who confirm their self-views and whose perceptions align with their self-concept, even if that self-concept is negative (De La Ronde & Swann, 1998; Swann, 2011). This theory suggests people prefer partners who agree with one’s self-views more so than being perceived in an overly positive, non-verifying manner. Evidence for self-verification may seem at odds with the positive illusions literature as the former suggests accurate perceptions are beneficial for relationships whereas the latter favors partner idealization. However, the two are not mutually exclusive, as partners can be both accurate and biased at the same time. Indeed, research on partner perceptions distinguishes between *tracking accuracy* and *directional bias* (or *mean-level bias*; e.g., Fletcher & Kerr, 2010; West & Kenny, 2011), demonstrating that people can simultaneously hold accurate and biased judgments toward a partner. In the context of daily relationship perceptions, for example, multiple judgments of a partner’s attributes over time can correspond generally to a partner’s own self-appraisals (tracking accuracy) over that same period while also being systematically biased in a positive direction (i.e., directional or mean-level bias): Alex may correctly recognize when Jesse engages in acts of love, care, or validation on a given day (i.e., tracking accuracy) but may tend to consistently “see” more (or less) than Jesse in fact reports (i.e., directional bias). Both processes, in turn, have been found to be associated with higher levels of relationship satisfaction (Fletcher & Kerr, 2010).

## Current Perspectives on Bias for Partner Interaction Attributes

A second key finding emerging from meta-analytic research on partner perceptions is that individuals exhibit different patterns of bias depending on the domain of judgment. Specifically, people tend to exhibit positive mean-level bias (i.e., overestimation bias) for judgments of a partner’s personality traits, relationship memories, reading a partner’s thoughts, or relationship forecasting; in turn, positively biased judgments in these domains predict higher relationship satisfaction (Fletcher & Kerr, 2010). Yet, the same research showed that individuals tend to exhibit *negative* mean-level bias (i.e., underestimation bias),^1^ when it comes to relationship judgments of a partner’s behaviors, attitudes, or beliefs focused on the connection between the self and the partner (i.e., *interaction attributes*; Fletcher & Kerr, 2010). Thus, when judging a partner’s daily relationship behaviors, such as their expressions of love, people tend to exhibit an *under*estimation bias, perceiving less than is actually the case (and correspondingly, people exhibit overestimation bias for “negative” interaction attributes, such as criticism).

A prevailing explanation for this opposing pattern draws on error management theory, an evolutionary account of social judgment and decision-making which suggests that cognitive processes have been shaped by evolutionary pressures to minimize the costs of errors in certain situations (Haselton & Buss, 2000). Given the importance of long-term romantic bonds for human survival (Gonzaga & Haselton, 2008), an error management perspective would indicate that romantic partners’ judgments may be biased in a particular direction (e.g., underestimation vs. overestimation) to the extent that such a bias might help sustain the relational bond. For judgments made specifically for partner interaction attributes (e.g., perceived partner love), underestimation would constitute the less costly error to make since individuals who underestimate would be able to respond more readily to potential relationship threats—such as a partner’s waning love—through engaging in greater relationship maintenance efforts. In turn, an overestimation bias would be a more costly error to make as such a bias might lead to relationship complacency and less efforts to strengthen the bond with one’s partner at critical times (Fletcher & Kerr, 2010). In contrast, for non-interaction attributes of a partner (e.g., physical attractiveness), an error management perspective would not predict similar patterns of bias as these attributes do not centrally pertain to how close and connected partners feel with one another (i.e., overestimating how attractive a partner is would be less directly relevant for maintaining the relationship). Taken together, research is currently guided by the view that individuals may “see the glass as half-empty” specifically for a partner’s interaction attributes and that such a bias is adaptive for relationships. Yet, limited research to date has sought to examine how underestimation of interaction attributes may be linked with relational benefits for *both* partners. Only a few studies have tested the dyadic effects of directional bias for certain interaction attributes, such as sacrifice motives and sexual rejection behaviors, and findings have not painted a clear picture in line with an error management perspective (e.g., Dobson et al., 2022; LaBuda & Gere, 2021; Muise et al., 2016).^2^ Thus, we aimed to address this open question by examining romantic partners’ perceptual accuracy for a prototypical interaction attribute in relationships: daily compassionate love behaviors.

## Compassionate Love Behaviors

A key feature underlying the multitude of positive relationship behaviors in the literature is that they principally center on fostering the relational bond between intimate partners. Thus, they fall under Fletcher and Kerr’s (2010) definition of interaction attributes which encompass behaviors, attitudes, and/or beliefs that speak to the interdependence within the relationship. To this end, we focused on expressions (and perceptions) of compassionate love in the present work as they represent a prototypical type of interaction attribute. While various typologies have guided the scientific study of love over the years (see Reis & Aron, 2008), compassionate love reflects a form of altruistic, caring love that emphasizes genuine concern for the other’s welfare, more so than other types of love, such as romantic or passionate love (Berscheid, 2010; Fehr et al., 2014; Underwood, 2009). Compassionate love behaviors are actions that are taken to promote flourishing within the relationship and reflect the mutual understanding and shared respect between partners; importantly, they are intrinsically relational in nature. Thus, compassionate love behaviors are *particularly* representative of capturing the interdependence between two relationship partners, a defining feature of interaction attributes (Fletcher & Kerr, 2010).

## Overview of the Present Research

In two daily experience studies of romantic couples, we asked whether people are accurate and/or biased in perceiving their partner’s compassionate love behaviors (hereinafter simply referred to as “CL”). We drew on distinct theoretical perspectives to inform how biased perceptions of CL may be associated with relationship outcomes. Of key interest was focusing on the dyadic context underlying perceptions of CL, and extending prior work using the Truth and Bias Model (T&B; West & Kenny, 2011) and Dyadic Response Surface Analyses (DRSA; Schönbrodt et al., 2018).

### T&B and DRSA Overview

Across studies, we conducted Truth and Bias analyses (T&B; West & Kenny, 2011) to examine patterns of accuracy and bias in people’s perceptions of their partner’s compassionate love. In T&B, perceivers’ ratings of their partner were compared with their partners’ actual rating to separately test the effects of tracking accuracy (e.g., the association or correlation between Alex’s perceived CL and Jesse’s reported CL) and directional bias (e.g., the degree to which Alex’s perceptions of Jesse’s CL are consistently more positive or negative than Jesse’s ratings). Here, we also included perceivers’ ratings of their own CL in our models to examine and account for the effect of assumed similarity (Kenny & Acitelli, 2001). The assumed similarity is another type of bias in which individuals’ perceptions of their partner are influenced by their own behaviors (e.g., how much Alex projects his own enacted CL onto his perceptions of Jesse’s CL). We thus assessed tracking accuracy, directional bias, and assumed similarity for perceptions of partner CL within a T&B framework. In line with standard approaches to studying dyadic relationship processes and their impact on *both* partners’ outcomes (i.e., the actor-partner interdependence model or APIM; Kenny et al., 2006), we also examined whether individuals’ accurate and biased perceptions are associated with their own satisfaction (i.e., *actor* effects) as well as their partner’s satisfaction (i.e., *partner* effects). Central to the research was testing the effects of directional bias (vs. tracking accuracy and assumed similarity) on actor and partner satisfaction.

Study 2 used DRSA to test whether certain combinations of biased perceptual patterns within dyads may be associated with greater relationship satisfaction. DRSA is specifically tailored to answer questions about whether satisfaction is higher or lower depending on whether partners exhibit similar or dissimilar directional biases (Schönbrodt et al., 2018). Yet, no research to our knowledge has sought to test whether couples are more (or less) satisfied if both partners exhibit overestimation bias, if both partners exhibit underestimation bias, if partners exhibit contrasting biases, or some another distinct dyadic pattern. Overall, three central aims guided the current research:

### Research Aim 1: Key Components of Interpersonal Accuracy

The first aim was to replicate prior research documenting biased patterns of judgment for interaction attributes by examining partners’ daily CL perceptions and behaviors. In light of past theory and empirical studies (Fletcher & Kerr, 2010), as well as robust effects of tracking accuracy and assumed similarity identified for similar interaction attributes in past work, we predicted that individuals would demonstrate significant underestimation bias, significant tracking accuracy, and significant assumed similarity for their partner’s CL behaviors.

### Research Aim 2: Interrelationships in Partners’ Directional Bias

The second aim was to examine the extent to which biased perceptions are interrelated between partners. Although questions of similarity and complementarity are central to relationship science (e.g., Dryer & Horowitz, 1997; Markey & Markey, 2007; Montoya et al., 2008), no research to our knowledge has yet explored whether partners tend to be similar or dissimilar in their biased perceptions. Thus, we focused on the potential dyadic links between partners’ biased perceptions of CL, and whether underestimation bias in one partner tends to be associated with an under- or overestimation bias in the other partner. We assessed this question in an exploratory manner given no established theory to inform hypotheses.^3^

### Research Aim 3: Associations With Relationship Satisfaction

The third aim was to examine how biased perceptions in a particular direction are associated with relationship satisfaction. We used both T&B (Studies 1 and 2) and DRSA (Study 2) as each addresses the question in distinct ways. With T&B, we tested how each of the three components of interpersonal accuracy was associated with actors’ and partners’ satisfaction at the daily level, with a specific focus on the effects of directional bias. Drawing on the prevailing error management account of biased perceptions for interaction attributes (Fletcher & Kerr, 2010), we expected that an underestimation bias for partner CL behaviors would be associated with higher relationship satisfaction. However, prior theory and research are unclear as to whether underestimation of CL is expected to predict improved relationship outcomes for both actors and partners equally. Among the few empirical studies that speak to this topic, underestimation of interaction attributes was associated with more negative relationship outcomes for the self (e.g., Dobson et al., 2022; LaBuda & Gere, 2021) yet more positive outcomes for one’s partner (e.g., LaBuda & Gere, 2021; Muise et al., 2016). T&B analyses were thus exploratory, with reason to expect that directional bias may not be linked with actors’ and partners’ satisfaction in a similar manner. To address the third aim, we additionally used DRSA in Study 2 to examine how both partners’ directional bias scores *combine* to predict relationship satisfaction. That is, we tested how both partners’ directional biases—considered together—predict relationship satisfaction in dyads (e.g., is it optimal if both partners tend to underestimate or overestimate?). We expand on our DRSA approach in the introduction to Study 2.

## Study 1

Study 1 consisted of a 2-week daily experience study that measured daily CL behaviors in a sample of mixed-sex and same-sex couples. We examined partner perceptions for CL as well as their associations with satisfaction. DRSA was not conducted in this study as the sample size was insufficiently powered for this method (see OSM).

### Method

Analyses were conducted in R. All materials (e.g., syntax, output, data, and preregistration details for studies) are available on OSF at https://osf.io/7sgye/).

#### Participants and Procedure

Fifty-nine couples were recruited using online ads (e.g., Craigslist, Kijiji) for a study on couples’ relationship experiences over time. Interested participants contacted researchers via email, and a trained research assistant phoned each couple with both partners present to confirm eligibility. Couples were required to be living together and fluent in English to participate. Three couples were removed as data integrity checks suggested one person completed both couple members’ surveys. The final sample consisted of 56 couples (53 mixed-sex, 3 same-sex) comprised of 53 men and 59 women. Participants had a mean age of 25 years (*SD* = 6, range = 18–47) and a mean relationship length of 3 years (range = 2–10).^4^ Ethnicity information was not collected. Participants first completed a 30-min background survey, then received daily surveys for 2 weeks; they also completed longer weekly surveys which were not used for the current research. Participants were compensated up to US$42 for completing all surveys in the study. Sample attrition was low: on average, participants completed 13.08 out of 15 diaries, and 2.83 of 3 weekly surveys.

#### Measures

##### Compassionate Love

Each day, participants rated on a 1 to 7 Likert-type scale the extent to which they and their partner engaged in 21 positive relationship behaviors (Joel et al., 2022; 1 = *not at all*, 7 = *very much*). Ten items were aggregated as composites of compassionate love behaviors similar to prior conceptualizations by Reis and colleagues (2014). Items included: “Listened attentively when I talked to him or her,” “Been respectful of my opinions and perspectives, “Told me that he or she appreciates me,” “Complimented me,” “Noticed when I was upset or down,” “Shown concern for my feelings and emotions,” “Remembered important or meaningful things that I told him or her,” “Showed support for my interests or projects,” “Shared things that were on his or her mind with me,” and “Told me that he or she loves me.” Participants rated the extent to which they (and their partner) engaged in these same behaviors with the prompt, “How much did you engage in the following relationship behaviors today?” Both own (*M* =5.70, *SD* = 1.21, *Rc* = .88) and perceived partner’s (*M* =5.53, *SD* = 1.35, *Rc* = .92) compassionate love behaviors were computed.

##### Relationship Satisfaction

Relationship satisfaction was measured each day on a 1 to 7 Likert-type scale (1 = *strongly disagree*, 7 = *strongly agree*) with a one-item measure (i.e., “*Today, I felt satisfied with our relationship*,” *M* =5.96, *SD* = 1.33).

### Data Analysis

Truth and Bias models tested the degree to which individuals are accurate and biased in their daily judgments of their partner enacting compassionate love behaviors. In these models, perceivers’ (i.e., the persons making judgments) ratings of their partner are compared with their partner’s actual ratings. As noted by West and Kenny (2011), in cases where individuals make multiple partner judgment ratings over several days, it is appropriate to person-center individuals’ judgments on their partner (i.e., center on the partner’s average report across days).^5^ This centering strategy means that the intercept represents the degree of *directional bias* or the difference between the average of the partner’s self-reported degree of enacted CL and the average of the perceivers’ judgments of their partner’s CL. The slope of partners’ reported love behavior ratings on perceivers’ judgments indicates whether there is significant *tracking accuracy*; the slope of perceivers’ own enacted CL ratings on their judgments indicates whether there is significant *assumed similarity* (i.e., projection).

We conducted lagged analyses to test how T&B effects were associated with both partners’ daily relationship satisfaction the next day while controlling for the previous day’s satisfaction.^6^ In T&B, the perceiver’s judgment is treated as the outcome variable; thus, the main effects of next-day actor and partner relationship satisfaction were included as predictors in the model (to assess associations with directional bias) as well as their interaction terms with partners’ reported CL and with own enacted CL (to assess associations with tracking accuracy and assumed similarity, respectively). The previous day’s satisfaction was included as a control, and all satisfaction variables were centered on the grand mean. Dyads were treated as indistinguishable (Kenny et al., 2006) to retain the same-gender couples in the dataset and to maximize statistical power given the small sample size. T&B models were assessed as two-level cross-classified random-intercept multilevel models in which daily reports were crossed with the individual and dyad levels (Bolger & Laurenceau, 2013). Standardized effect sizes for estimates were not computed as there is no widely agreed-upon method of doing so for these models (Rights & Sterba, 2019).

### Results

#### Directional Bias, Tracking Accuracy, and Assumed Similarity

To address the first research aim, we found a significant underestimation of partner CL behaviors in relationships, as well as significant effects of tracking accuracy and assumed similarity, consistent with hypotheses (Table 1).

**Table 1.** Study 1 Directional Bias, Tracking Accuracy, and Assumed Similarity Effects.

Perceptions of partner’s compassionate love behaviors	*b*	95% CI	*SE*	*t*	*p*
Directional bias	–.171	[–.233, –.108]	.032	–5.347	<.001
Tracking accuracy	.192	[.148, .237]	.020	8.408	<.001
Assumed similarity	.878	[.838, .918]	.023	42.884	<.001

#### Within-Couple Association of Directional Bias

To address the second research aim, we specified the correlation between partners’ random intercepts in our models, as is standard in multilevel APIM analyses (Kenny et al., 2006). Conceptually, within a T&B framework, the estimate of each participant’s random intercept reflects their overall directional bias averaged across diary days, with more positive values indicating greater overestimation and negative values indicating greater underestimation. This correlation thus represents the within-couple association between partners’ directional biases. Results showed that partners’ directional bias scores were negatively correlated (*r* = –.159, confidence interval [CI_95%_] = [–.461, .177]). Actors who underestimated more had partners who overestimated more, although follow-up nested model comparisons determined this inverse correlation was not statistically significant when compared against a model with the within-couple correlation fixed to zero, χ²(1) = .855 *p* = .355.

#### T&B Associations With Satisfaction

The third research aim examined how directional bias is associated with daily actor and partner relationship satisfaction (effects of tracking accuracy and assumed similarity are also presented in Table 2). Results showed differential effects of directional bias for actor versus partner next-day satisfaction. Greater overestimation was associated with significantly higher next-day satisfaction for actors (*b* = .039, standard error [*SE*] = .020, *p* = .048), but associated with lower next-day satisfaction for partners, although this latter effect was non-significant (*b* = –.030, *SE* = .019, *p* = .120). Furthermore, greater tracking accuracy was significantly associated with lower partner satisfaction (*p* = .034).

**Table 2.** Study 1 T&B Associations With Satisfaction

Effects of T&B components	*b*	95% CI	*SE*	*t*	*p*
Directional bias	–.178	[–.247, –.108]	.036	–4.970	<.001
Actor satisfaction	.039	[.001, .078]	.020	1.980	.048
Partner satisfaction	–.030	[–.067, .008]	.019	–1.557	.120
Tracking accuracy	.137	[.083, .191]	.028	4.926	<.001
Actor satisfaction	.023	[–.013, .059]	.018	1.257	.209
Partner satisfaction	–.041	[–.079, –.003]	.019	–2.129	.034
Assumed similarity	.798	[.749, .848]	.025	31.626	<.001
Actor satisfaction	.014	[–.010, .037]	.012	1.121	.262
Partner satisfaction	–.003	[–.025, .019]	.011	–.276	.782

*Note.* Model controls for the previous day’s actor and partner satisfaction.

### Study 1 Brief Discussion

Study 1 found that people underestimate their partner’s CL yet also demonstrate tracking accuracy and assumed similarity. There was also initial evidence that partners’ directional biases are inversely related such that greater underestimation is related to greater overestimation among partners. Furthermore, there was evidence that directional bias was differentially associated with next-day’s actor and partner outcomes: underestimation predicted significantly lower actor satisfaction but did not significantly predict partner satisfaction. Considering the sample size limitations in this study, we sought to probe these effects further in a larger, higher-powered study, one which additionally enabled the use of DRSA to examine the effects of both partners’ directional bias on couples’ satisfaction.

## Study 2

In Study 2, we analyzed partner perceptions of daily compassionate love behaviors and their associations with satisfaction in a larger sample of mixed-sex couples who completed a 2-week daily diary study. Here, we also conducted DRSA to evaluate our competing hypotheses about what pattern of partners’ directional biases appears to optimally predict relationship satisfaction.

### DRSA Overview

DRSA is a technique ideally suited to test questions about whether partners are more satisfied when they are more similar versus dissimilar in their degree of overestimation or underestimation bias by incorporating both linear and curvilinear effects. Specifically, DRSA estimates a polynomial regression within an APIM framework to examine the linear and curvilinear combinations of how two persons’ predictor variables (i.e., directional bias) are associated with an outcome (i.e., relationship satisfaction; see Schönbrodt et al., 2018, for a review. In DRSA, effects are plotted as a response surface in three-dimensional space according to the a-parameters, which are tested for statistical significance (for a review, see Schönbrodt et al., 2018 and Shanock et al., 2010 as a comprehensive overview of response surface methodology is outside the scope of the current work).

**Figure 1.**
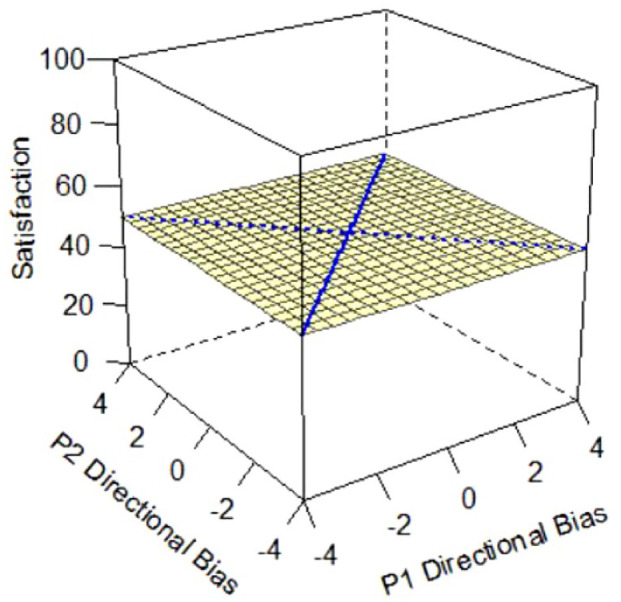
Example Response Surface Plot Using Current Study Variables.

Applied to the current research, and as shown in Figure 1, X and Y axes depict each partner’s respective directional bias score as predictor variables; scores of 0 indicate perfect accuracy (i.e., complete absence of any degree of bias), and more positive and negative values indicate greater degrees of over- or underestimation bias, respectively. The response surface reflects the expected values of the outcome variable (satisfaction) represented on the Z-axis, predicted from all possible combinations of the two predictors. Figure 1 depicts a response surface in which all combinations of partners’ directional bias scores predict the same level of satisfaction.

Here, we focus on statistical details regarding the a-parameters that directly inform our research question and are central to interpreting the competing hypotheses outlined in Table 3 below and represented by Figures 2a-e. The surface value a1 tests levels of satisfaction on the slope of the line of congruence (i.e., where couples perfectly match on the directional bias, Figure 1 solid line). Drawing on established interpretative guidelines in the literature (Humberg et al., 2019), if only a1 is significant and negative, it indicates that couples with higher directional bias (greater overestimation) are less satisfied than couples with lower directional bias (greater underestimation; Model 1: Dyadic error management hypothesis). A significant and positive a1 in isolation indicates those same couples are more satisfied than couples with lower directional bias (Model 2: Dyadic positive illusions hypothesis). The surface value a2 tests the curvature of the line of congruence: a significant and negative a2 in isolation would reflect that the highest levels of satisfaction are along the line of *in*congruence; thus, couples who match at more extreme levels (i.e., high overestimators or high underestimators) are less satisfied than couples who both exhibit less directional bias or couples who mismatch in directional bias (i.e., overestimators paired with underestimators; Model 3: Dyadic complementarity hypothesis). Similar to a1 and a2, respectively, the surface values a3 and a4 test for the slope and curvature of satisfaction along the line of *in*congruence (i.e., where couples perfectly *mis*match on their directional bias, Figure 1 dotted line). Thus, a significant a3 would indicate whether partners who *mis*match in a particular direction (e.g., male partner overestimates, female partner underestimates) are more or less satisfied compared with partners who mismatch in the other direction (e.g., male partner underestimates and female partner overestimates). A significant a4 would indicate whether couples with more extreme levels of mismatching (e.g., high overestimator-underestimator couples) are more or less satisfied compared with couples at more moderate levels of mismatching on their directional bias scores (e.g., low bias couples). A significant a4 is a necessary but not sufficient condition for testing if couples are most satisfied when they are more similar in directional bias, regardless of the direction or level of the bias (Humberg et al., 2019; Model 4: Dyadic similarity hypothesis). The surface value a5 represents the degree to which the “ridge” of the response surface is shifted away from the line of congruence, which helps in evaluating similarity effects (Schönbrodt et al., 2018). Finally, a significant, negative a2 and a4 together would indicate that couples at more moderate levels of directional bias (i.e., low-biased couples) are more satisfied than couples who match or mismatch at more extreme levels of directional bias [Model 5: Dyadic self-verification hypothesis].

**Table 3.** Overview of Competing DRSA Hypotheses.

**Model 1. Dyadic error management hypothesis** An error management hypothesis suggests greater “negative” bias is associated with benefits in relationships. Accordingly, couples may be most satisfied when both partners underestimate, compared to all other types of couples (i.e., two overestimators or couples with opposing directional biases). *Key supporting DRSA parameters: negative, sig. a1; non-sig. a3, a4, and a5.*	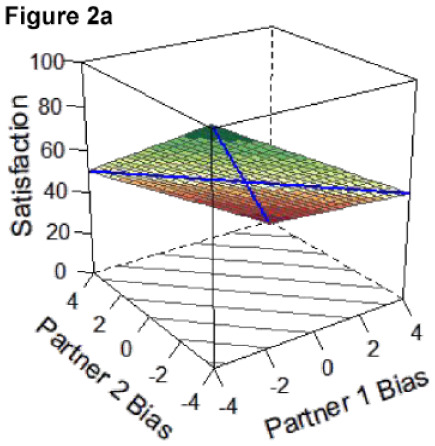
**Model 2. Dyadic positive illusions hypothesis** A positive illusions perspective suggests greater positive bias is associated with greater benefits in relationships. Accordingly, couples may be most satisfied when both partners overestimate, compared to all other types of couples. *Key supporting DRSA parameters: positive, sig. a1; non-sig. a3, a4, and a5.*	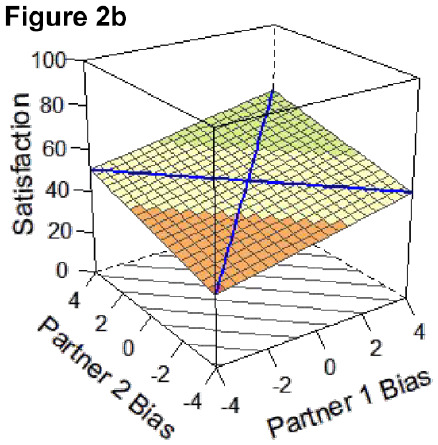
**Model 3. Dyadic complementarity hypothesis** A complementarity perspective draws on mixed evidence suggesting partner complementarity is associated with greater benefits in relationships. Accordingly, couples may be most satisfied when partners exhibit opposing patterns of directional bias; that is, when one partner overestimates and the other underestimates at similar degrees.^7^ *Key supporting DRSA parameters: negative, sig. a2; non-sig. a3 and a4.*	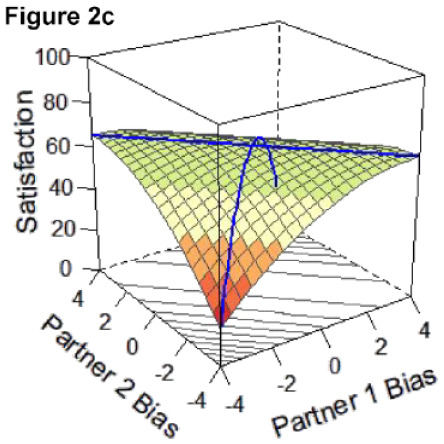
**Model 4. Dyadic similarity hypothesis** A similarity perspective draws on the robust literature suggesting partner similarity is associated with greater benefits in relationships. Accordingly, couples may be most satisfied when both partners are more similar in their directional bias. *Key supporting DRSA parameters: negative, sig. a4; non-sig. a3 and a5.*	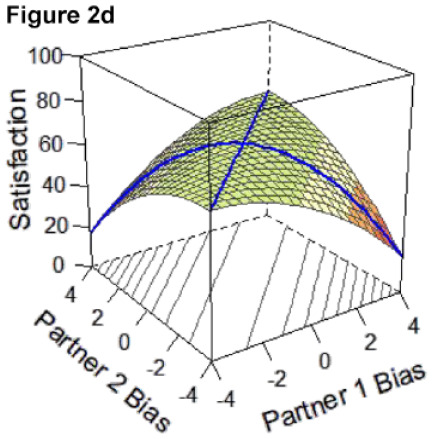
**Model 5. Dyadic self-verification hypothesis** A self-verification perspective suggests fewer discrepancies between self- and partner-ratings (i.e., less biased perceptions) are associated with greater benefits in relationships. Accordingly, couples may be most satisfied when both partners exhibit less biased perceptions in either direction (i.e., overestimation or underestimation). *Key supporting DRSA parameters: negative, sig. a2 and a4; non-sig a5.*	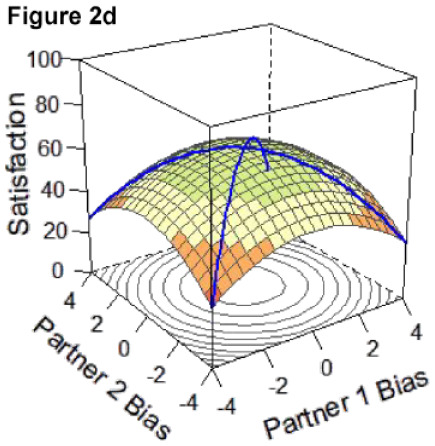

*Note.* DRSA = Dyadic Response Surface Analysis.

### DRSA Hypotheses

We set up competing DRSA hypotheses about how two partners’ directional biases combine to predict greater relationship satisfaction (Table 3). Hypotheses drew on theoretical perspectives on partner perceptions outlined earlier (e.g., positive illusions, self-verification, and error management). Hypotheses also encompassed potential effects of similarity or complementarity in partners’ directional biases insofar as DRSA is primed to test for effects of *congruence*: that is, whether matching or mismatching between two predictor variables is associated with higher values on an outcome (Humberg et al., 2019). Indeed, research has long been interested in whether similarities between partners (e.g., on personality traits, attitudes, and values) predict better relationship outcomes (e.g., Luo, 2017; Montoya et al., 2008); however, findings have been mixed (e.g., Gaunt, 2006; Watson et al., 2004). Likewise, studies have examined whether complementarity—in which partners have opposing characteristics—predicts better relationship outcomes (e.g., Markey & Markey, 2007). Complementarity effects center on the idea that partners may benefit by balancing each other’s strengths and weaknesses (e.g., Dryer & Horowitz, 1997; Vohs et al., 2011). Although evidence for complementarity effects has been limited (e.g., Finkel et al., 2012; Klohnen & Mendelsohn, 1998), some studies suggest complementarity may have benefits when it comes to partners’ goal-pursuit strategies (Bohns et al., 2013) or in longer-term relationships (Shiota & Levenson, 2007). Taken together, five competing DRSA hypotheses were proposed, with DRSA statistical parameters to support each model determined based on established guidelines for interpreting response surface parameters and effects of mis/matching (Humberg et al., 2019; Schönbrodt et al., 2018). Table 3 outlines these criteria, alongside a prototypical graphical depiction of the pattern supporting each hypothesis.

### Method

#### Participants and Procedure

Participants consisted of 175 mixed-sex newlywed couples (175 men, 175 women) in North America recruited from various sources (e.g., bridal shows, social media, online forums) as part of a larger study on everyday behavior in early marriage.^8^ Couples have married an average of 7 months (*SD* = 4, range = 1–16), had known each other an average of 6 years (*SD* = 4, range = 7 months–22 years), and had a mean age of 28 years (*SD* = 5, range = 18–50). Participants were 70% Caucasian, 5% Hispanic, 7% African American, 12% Asian, and 6% Multiracial or Other. Couples were screened by survey and phone and excluded if they: lived apart from each other; were older than 50; reported domestic violence; or had been hospitalized for emotional disorders or abused alcohol or drugs. Of 214 eligible couples who passed the screening procedure, 175 participated in the 2-week daily diary study. Couples received up to US$100 for completion of daily diaries in addition to a raffle entry for four US$100 USD prizes. All data were collected online. After an initial questionnaire that included demographic information (and other measures not relevant to this research), participants received a daily survey over email for 2 weeks from 7 pm to 9 am the next day. Participants completed an average of 13.2 out of the 14 daily diaries.

#### Measures

##### Compassionate Love

Participants reported on 10 daily compassionate love behaviors (Reis et al., 2014), with parallel items capturing self and partner behavior (e.g., *“I said or did something to show that I value my partner,” “My partner willingly put my goals or wishes ahead of his or her own”*). Participants reported whether each behavior occurred as a binary response (1 = *yes*, 0 = *no*). Composites representing both self-reported and perceived partner reports of compassionate love were computed by averaging across the 10 items and then multiplying this value by 10 (*M* = 6.04, *SD* = 3.14).

##### Relationship Satisfaction (Baseline and Daily)

At study baseline, participants completed the 16-item Couple Satisfaction Index (Funk & Rogge, 2007; scale range 0–81; *M* = 69.59, *SD* = 9.49). Daily relationship satisfaction was measured by averaging three items (*“Today our relationship was terrible/terrific,” “Today, I felt close and connected to my partner*,” *“Today, I enjoyed our time together”*) rated on 1 to 7 Likert-type scales (1 = *not at all*, 7 = *a great deal; M* = 5.71, *SD* = 1.16, *Rc* = .84).

### Data Analysis and Results

#### T&B Analyses

T&B analyses were conducted as a higher-powered replication of Study 1. A test of distinguishability found that dyads were not empirically distinguishable (*p* = .052), thus we ran indistinguishable models as in Study 1.^9^ Consistent with hypotheses and Study 1 results, there was a significant underestimation of partner CL as well as significant tracking accuracy and assumed similarity (Table 4).

**Table 4.** Study 2 Directional Bias, Tracking Accuracy, and Assumed Similarity Effects.

Perceptions of partner CL	*b*	95% CI	*SE*	*t*	*p*
Directional bias	–.251	[–.364, –.138]	.058	–4.347	<.001
Tracking accuracy	.287	[.260, .315]	.0134	20.154	<.001
Assumed similarity	.551	[.524, .577]	.0141	41.153	<.001

*Note.* CI = confidence interval

**Table 5.** Study 2 T&B Associations With Actor-Partner Satisfaction

Effects of T&B components	*b*	95% CI	*SE*	*t*	*p*
Directional bias	–.274	[–.382, –.166]	.055	–4.34	<.001
Actor satisfaction	.089	[.009, .169]	.041	2.184	.029
Partner satisfaction	–.118	[–.198, –.039]	.041	–2.903	.004
Tracking accuracy	.243	[.213, .273]	.015	15.75	<.001
Actor satisfaction	–.003	[–.030, .025]	.014	–.208	.836
Partner satisfaction	.023	[–.004, .050]	.014	1.688	.092
Assumed similarity	.431	[.402, .459]	.015	29.501	<.001
Actor satisfaction	.003	[–.016, .023]	.010	.340	.262
Partner satisfaction	.007	[–.012, .027]	.010	.728	.782

*Note.* Model controls for the previous day’s actor and partner satisfaction.

#### Within-Couple Association of Directional Bias

As in Study 1, there was a negative correlation between partners’ directional bias (*r* = –.506, CI_95%_ = [–.631, –.355]): the more one partner overestimated, the more the other partner underestimated. Unlike Study 1, this value was significant when compared with a model in which the within-couple correlation was fixed to zero, *χ*²(1) = 35.088, *p* < .001.

#### T&B Associations With Daily Satisfaction

As in Study 1, we included the main effects of next-day’s actor and partner satisfaction in our models and their interactions with directional bias, tracking accuracy, and assumed similarity, controlling for the previous day’s satisfaction. Again, we found differing effects of directional bias on actor and partner satisfaction. Greater underestimation of partner CL predicted significantly lower actor satisfaction (*b* = .089, *SE* = .041, *p* = .029). In contrast, and consistent with the direction of effects in Study 1, underestimation predicted significantly higher satisfaction for partners (*b* = –.118, *SE* = .041, *p* = .004).

#### Dyadic Response Surface Analysis

DRSA assessed how partners’ directional biases combine to predict relationship satisfaction.^10^ To test combinations of partners’ directional bias as predictor variables in DRSA, we extracted a directional bias score for each individual and their partner, consistent with similar approaches in prior work (e.g., Stern & West, 2018). Specifically, we extracted the random intercept estimates for each individual participant based on their (and their partner’s) daily variables in the T&B models. These estimates effectively represent trait-like scores, in which positive values indicate the participant was an overestimator, while negative values indicate the participant was an underestimator. After extracting these directional bias scores, we ran DRSA using both partners’ scores as predictor variables to see how within-couple patterns of directional bias predict baseline satisfaction. To further isolate the effects of directional bias, we controlled for both partners’ mean levels of enacted CL across the diary in DRSA models.

Analyses were conducted in R with structural equation modeling following procedures outlined in Schönbrodt et al. (2018). In line with standard practice, we tested whether results were distinguishable by gender, applying equality constraints by participant gender and comparing models using *χ*^2^ likelihood ratio tests. A gender-constrained model did not significantly worsen model fit, *χ*^2^(5) = 6.810, *p* = .235; thus, dyads were treated as indistinguishable, and results are described with reference to actors and partners (see online supplemental material). Final models were computed by bootstrapping standard errors and p values with 10,000 replications.

There was sufficient variability in directional bias scores across the sample to warrant the use of DRSA. Of the 350 total participants (175 couples), 190 (56%) had negative directional bias scores, and 154 (44%) had positive directional bias scores. Of note, a non-zero directional bias score does not indicate a significant under- or overestimation bias on behalf of a participant, but simply the degree of their orientation toward one of these patterns (similarly, values closer to 0 would reflect an orientation toward perfect accuracy). Furthermore, 74% of couples were discrepant in their directional bias scores (determined by half a z-score unit difference; Shanock et al., 2010); 26% had similar scores, thus exceeding the threshold to test for curvilinear effects inherent to Models 3 to 5 (i.e., > 10% discrepancies in predictors; Shanock et al., 2010). We evaluated DRSA parameters (i.e., regression coefficients b1-b5; response surface coefficients a1-a5) following standard guidelines for interpreting response surface parameters and effects of dis/similarity (e.g., Humberg et al., 2019; Schönbrodt et al., 2018).

#### DRSA Results

The resulting DRSA model is shown in Table 6. DRSA best supported the Dyadic Complementarity Hypothesis (Model 3, Figure 2c in Table 3). That is, satisfaction was highest among couples in which one partner was an underestimator and the other an overestimator (evidenced by a negative, significant *a*2 coefficient and nonsignificant *a*3 and *a*4 coefficient). Second, contrary to the Dyadic Error Management Hypothesis and Dyadic Positive Illusions Hypothesis, couples were neither more nor less satisfied when both partners exhibited stronger underestimation or overestimation bias (evidenced by a nonsignificant *a*1). A significant a5 parameter suggested that the “ridge” of the response surface does not fall directly along the line of congruence (Schönbrodt et al., 2018).

**Table 6.** Polynomial and Response Surface Slope Coefficients for Relationship Satisfaction

Polynomial coefficients	Relationship satisfaction			Response surface
	*Estimate*	*SE*	*p*	
*b0*	71.564	.646	<.001	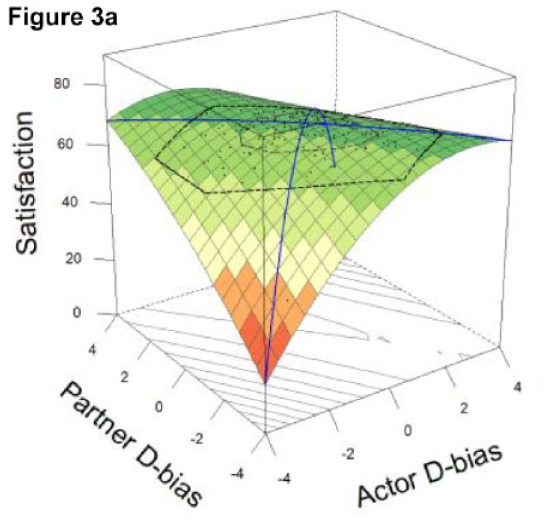
*b1 (X)*	1.175	.696	.092	
*b2 (Y)*	.966	.704	.170	
*b3 (X* ^2^ *)*	–1.195	.283	<.001	
*b4 (XY)*	–1.401	.470	.003	
*b5 (Y* ^2^ *)*	–.357	.320	.265	
Response surface parameters				
α*1*	2.141	1.342	.111	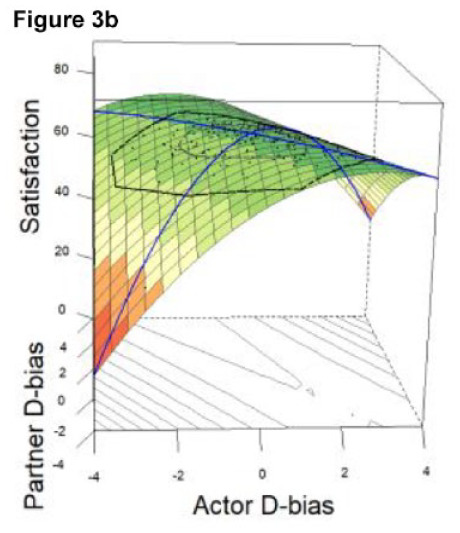
α*2*	–2.953	.898	.001	
α*3*	.209	.401	.603	
α*4*	–.152	.336	.652	
α*5*	–.838	.356	.018	

*Note.* X = Actor’s directional bias, Y = Partner’s directional bias (positive and negative values correspond to overestimation and underestimation, respectively). Partners’ levels of enacted CL were included as covariates. Graphical representation of the final model depicted in Figure 3 and Figure 3a (alternate view). As there is typically high collinearity between predictor variables in couples’ data, variance inflation factors (VIFs) were computed for each DRSA model predictor; all VIFs were <4.0 (see OSM).

### Study 2 Discussion

In Study 2, we tested the same effects as in Study 1 using a higher-powered sample and additionally conducted DRSA to assess the dyadic effects of partners’ directional biases on satisfaction. Consistent with Study 1, there was a significant effect of underestimation for partner’s daily compassionate love behaviors as well as robust effects of tracking accuracy and assumed similarity. Partners’ directional biases were again inversely related (overestimators tended to pair with underestimators), and this association was significant in Study 2. Furthermore, underestimation was associated with significantly lower relationship satisfaction for actors but greater satisfaction for partners.

Overall, DRSA results revealed limited evidence that underestimation of partner’s compassionate love behaviors predicts relationship satisfaction unequivocally. Rather, underestimation predicted greater satisfaction within the context of dyadic complementarity; that is, the highest levels of relationship satisfaction were represented by couples along the line of *in*congruence, in which one partner underestimated but the other overestimated, not couples who exhibited similar levels of overestimation or underestimation. Alternatively, couples who matched at more extreme levels (i.e., high overestimators or high underestimators) were less satisfied than couples who mismatched in directional bias (i.e., overestimators paired with underestimators). Thus, there was limited evidence to support the dyadic positive illusions hypothesis, dyadic self-verification hypothesis, or dyadic similarity hypothesis; instead, the dyadic complementarity hypothesis was best supported. Arguably, these findings do not unequivocally refute propositions derived from error management theory or positive illusions. Rather, they may simply show that the purported benefits of underestimation and overestimation for interaction attributes such as CL are best understood within a dyadic framework, rather than looking at one partner’s directional bias. This view is consistent with the transactive goal dynamics theory (Fitzsimons et al., 2015), reviewed in the general discussion. To our knowledge, this is the first research to simultaneously assess both partners’ perceptual biases together as predictors of relationship outcomes.

To enhance confidence in the current findings, we conducted several auxiliary analyses to address potential alternative explanations. First, we considered the robust effects of assumed similarity identified in both studies, and whether assumed similarity could account for the inverse correlation in partners’ directional biases. That is, inversely associated biases between partners might not reflect true complementarity in how partners perceive each other’s CL, but rather, differences in their enacted levels of CL. For instance, one partner’s underestimation may be driven by their enacting lower levels of CL, which they project onto their partner, and which is then negatively correlated with the partner’s overestimation (driven by their higher level of enacted CL). To an extent, our analyses controlled for this possibility. The assumed similarity variable in our T&B models was defined as the actor’s reported level of enacted CL centered on their partner’s reports of enacted CL. By including this variable in the model, the derived values for directional bias and the within-couple correlation control for the effects of enacted love. The inverse correlation finding is thus robust against this alternative. Nevertheless, when we excluded the assumed similarity variable from the model, the within-dyad correlation between partners’ directional bias was stronger in Study 1 (*r* = –.889) and Study 2 (*r* = –.839), suggesting assumed similarity contributes to an inverse correlation between directional biases. However, it is important to recognize that our main result rules out the possibility of assumed similarity driving the evidence for complementarity.

Second, we sought to explore whether the opposing pattern of effects of directional bias on actor and partner satisfaction (i.e., underestimation generally predicting lower actor satisfaction but greater partner satisfaction) may be explained by the inverse association between partners’ biases. For instance, our results show that actor underestimation is associated with partner overestimation, which in turn predicts greater partner satisfaction; underestimation may be linked to greater partner satisfaction insofar as it predicts a partner overestimating more. To assess this possibility, we re-ran our satisfaction models fixing the correlation between partner random effects to zero (i.e., the within-dyad random intercept correlation). This enabled a test of the associations between directional bias and actor-partner daily satisfaction assuming partners’ directional biases are not inversely related. All of the reported results held, suggesting these effects are not driven by partner complementarity in directional bias (see online supplementary material).

## General Discussion

The current research examined people’s perceptual accuracy for their partner’s compassionate love and how biased perceptions are associated with relationship satisfaction. We focused on daily compassionate love behaviors as they represent a prototypical positive relationship behavior central to established theories of optimal relationship functioning and relationship cognition. Specifically, we employed a dyad-centered approach to advance current knowledge on how biased perceptions of partner interaction attributes (Fletcher & Kerr, 2010) operate both within and between partners. We replicated findings across two studies using different measures of compassionate love, informing current gaps in the literature regarding patterns of directional bias, the interrelationship between partners’ biased perceptions, and their dyadic links with relationship satisfaction. Our analyses used two innovative statistical techniques, the T&B Model and DRSA, to derive more complex insights than prior methods allowed.

### Directional Bias, Tracking Accuracy, and Assumed Similarity

We used T&B analyses to differentiate between the relative effects of tracking accuracy, directional bias, and assumed similarity, all three of which are central constructs in this literature. Consistent with hypotheses, we found that people significantly underestimate their partner’s compassionate love behavior while also accurately tracking these behaviors. These effects of underestimation and tracking accuracy for compassionate love were consistent across studies and converge with prior findings on perceptions of partner interaction attributes (Fletcher & Kerr, 2010). We also found robust evidence that people project their own enacted compassionate love behaviors onto their perceptions of their partner (i.e., assumed similarity), consistent with prior work demonstrating projection in communal responsiveness (e.g., Lemay et al., 2007; Lemay & Clark, 2008). Given the robust effect of assumed similarity identified in our models, we also considered its role as a potential confound for the patterns of directional bias evidenced. Auxiliary analyses indicated that assumed similarity effects heightened but did not completely account for the inverse association between partners’ directional bias.

### Differential Effects of Directional Bias for Actor-Partner Satisfaction

Notably, the current dyadic approach found differential patterns in how underestimation was associated with satisfaction in couples. We initially asked whether it is better for couples to see the glass as half-full or half-empty when gauging their partner’s compassionate love. The answer, instead, appears to be both. Specifically, we found that an overestimation bias for partner CL (indicative of a positive illusions account) was associated with higher satisfaction for actors, whereas an underestimation bias (indicative of an error management account) was associated with higher satisfaction for partners. In this sense, both theoretical accounts can be interpreted as receiving partial support, insofar as the relationship benefits derived from each type of bias appear to depend on whose outcome is the subject of examination. Notably, these differential actor and partner effects align with findings from research examining partner perceptions for other interaction attributes (e.g., Dobson et al., 2022; LaBuda & Gere, 2021; Muise et al., 2016); however, research is needed to better understand the implications of differential actor and partner effects. Although we replicated the general pattern of effects in two studies, we note that the sample size for Study 1 was relatively small; among the key effects of interest in our T&B analyses, we may have been particularly underpowered to detect the partner effect of directional bias on satisfaction (see online supplementary material). Thus, we interpret this finding with caution. Also, DRSA was also limited to Study 2, and these findings warrant further replication across larger, more diverse samples.

### Complementarity Effects and Theoretical Explanations

The current findings provide novel evidence for patterns of complementarity in partners’ relationship perceptions. First, we identified an inverse association between partners’ directional biases at the person level: that is, greater overestimation by one partner was linked with greater underestimation by the other. To our knowledge, no previous work has considered how complementarity may underlie patterns of partners’ relationship judgments and inform key couple outcomes. This research is the first to explicitly impose a dyad-centered approach by exploring how both partners’ biased perceptions are interrelated as well as how they combine to predict relational satisfaction. To address this latter question, we used DRSA, examining several competing hypotheses based on prevailing notions within the partner perceptions literature. DRSA results provided support for the view that partners who demonstrated complementary directional biases were more satisfied than couples in which both partners were under- or overestimators.

These findings inform current theoretical perspectives governing the nature and impact of relationship judgments, particularly concerning perceptions of partner interaction attributes, a crucial component of optimal relationship functioning (Fletcher & Kerr, 2010). However, they also invite new questions about why complementary patterns predict greater satisfaction in couples. Although findings on complementarity (e.g., on partner traits) have been generally sparse, especially in recent years, the domain of partner perceptions may be one in which couples exhibiting complementarity are able to capitalize on the potential benefits (and simultaneously minimize the potential costs) associated with overestimation and underestimation bias. One possible explanation is that partners adjust to each other’s expressions of love to better establish their separate identities within the context of a close relationship (Charania & Ickes, 2007). Another possible explanation is that doing so provides specific advantages for optimal relationship functioning, with partners compensating for each other’s shortcomings. This would be consistent with transactive goal dynamics theory (Fitzsimons et al., 2015), which conceptualizes two partners not as distinct individuals but as complementary parts of a single self-regulating system in which they have shared goals and outcomes. When viewed from this perspective, complementarity in partners’ biased tendencies may reflect a form of interdependence that is functional. Holding differing cognitive biases could potentially help coordinate goal pursuit across various contexts and domains. Relationships comprised of both underestimation and overestimation may be able to capitalize on the advantages while also mitigating the costs associated with each of these biases. By contrast, if partners were to hold the same type of directional bias, it might be inefficient for maximizing *shared knowledge and motives*, in that when one partner contributes something to the relationship, the other does not need to do so (Wegner et al., 1991). For example, findings from the current studies lend credence to the idea that a successful relationship dynamic may be one in which partners are able to contribute different strengths to the relationship, such as when one partner’s overestimation helps promote positive sentiment in the relationship while the other’s underestimation helps in responding to potential problems.

Overall, the weighting of benefits and costs may fundamentally shift when examining couples as a dyadic unit rather than the individual experiences of each partner. If complementary directional biases facilitate couple satisfaction, this would suggest that researchers exert caution in their interpretations of how biased perceptions of partners may be adaptive for couples. It would also suggest important new directions for future empirical studies and theorizing. At the very least, the current findings provide compelling evidence that the impact of perceptual accuracy and bias involves a process that may be fundamentally dyadic in nature and they reinforce the value of adopting a dyadic framework.

### Limitations and Future Directions

The current research is limited in several ways. First, the data are correlational in both studies; thus, we cannot definitively confirm the causal direction of perceptual biases shaping partners’ satisfaction. Although it is possible that daily relationship quality leads to perceptual biases rather than the reverse, we controlled for the previous day’s relationship quality to bolster the inference that individuals’ biased perceptions of CL are followed by a change in their own and their partner’s relationship satisfaction (Bolger & Laurenceau, 2013). Furthermore, our conceptualization of bias consisted of treating the partner’s (i.e., target’s) self-report as the truth criterion, against which the judgment (of the perceiver) is evaluated. Although prior research commonly adopts a similar approach, different frameworks for determining truth criteria exist (e.g., West & Kenny, 2011). It is thus possible that an underestimation bias is a result of partners over-reporting their enacted CL behaviors. We consider this to be less plausible presently given that expressions of compassionate love behavior typically involve intentions of care and concern that would be inherently more accessible on the part of the provider (i.e., partner) versus the receiver (i.e., the perceiver). Future research could help rule out this possibility using lab-based studies of couples’ behavior with third-party observation. The research is also limited in that it focused on the directional bias for interaction attributes; thus, the results do not speak to the relational effects of bias for non-interaction attributes of partners. Furthermore, the current studies examined patterns of directional bias and their associations with partners’ relationship satisfaction but not partners’ relationship maintenance behaviors. The latter could better inform current explanations regarding the adaptive function of underestimation bias for partner interaction attributes, which suggests that the relational benefits of such bias are tied to the way that they spur relationship maintenance at critical times. Related to this, our findings broadly point to context as an important factor in determining when and for whom underestimation may be beneficial. One direction of future research is to identify the individual difference and relationship-specific factors related to biased perceptions of interaction attributes and the effects of complementarity in couples. For instance, an underestimation bias may prompt individuals to engage in relationship maintenance behavior to sustain partner regard, particularly in situations where relationship complacency or a partner’s waning love is a legitimate and warranted concern. Prior research suggests the benefits and costs of biased relationship cognitions can differ based on how unstable a relationship is, or when assessing relationship outcomes concurrently versus over time (McNulty et al., 2008). The current research was limited in that it centered on associations between underestimation and satisfaction at the daily and person levels. Assessing outcomes over a longer interval would provide important theoretical insights, and identify potential boundary conditions on the relational benefits associated with over- or underestimation biases (e.g., for partner interaction attributes). Furthermore, as most couples in both studies indicated being relatively satisfied, future research could examine the generalizability of findings when more variability in the outcome variable is present, such as in distressed or less satisfied couples.

Similarly, a key direction for future research is to directly test for context-dependent mechanisms (e.g., goal coordination) underlying the complementarity effects reported here to better understand when and why complementarity in partners’ biased perceptions might be beneficial. Rigorous testing of such mechanisms using a dyad-centered approach may be integral to advancing knowledge on key relationship processes.

## Supplemental Material

sj-docx-1-psp-10.1177_01461672231171986 – Supplemental material for Half Empty and Half Full? Biased Perceptions of Compassionate Love and Effects of Dyadic Complementarity Supplemental material, sj-docx-1-psp-10.1177_01461672231171986 for Half Empty and Half Full? Biased Perceptions of Compassionate Love and Effects of Dyadic Complementarity by James J. Kim, Harry T. Reis, Michael R. Maniaci and Samantha Joel in Personality and Social Psychology Bulletin

## Notes

1. Although Fletcher and Kerr (2010) call these “interaction traits,” we use the term “interaction attributes” to more broadly reflect that these do not encompass personality traits of partners.

2. A few studies have yielded somewhat inconsistent results for other attributes which would fall under this category (see OSM).

3. We are aware of one investigation on perceptions of partner support behaviors (Bar-Kalifa et al., 2016) in which partners’ directional biases were found to be inversely (i.e., negatively) related. Although we did not establish an a priori hypothesis, we considered a similar result to be plausible.

4. A priori power analyses were not conducted, but a sensitivity power analysis using the PowerLAPIM application (Lafit et al., 2022) based on the current sample size and study design determined we had 72% power to detect the minimum effect size observed among key effects in our T&B models (see OSM).

5. Analyses were also conducted using grand-mean centering with consistent results (see OSM).

6. We also conducted lagged analyses testing for the current-day’s satisfaction (controlling for the previous day’s satisfaction). Results were consistent (see OSM).

7. Of note, complementarity effects have been conceptualized in different ways in the literature. The current approach emphasizes the qualitative *pattern* of complementarity (mismatching across all levels whereby Y = −X) rather than the degree of complementarity (more versus less extreme levels of mismatching). See OSM for additional details.

8. These data have been used in a prior study on partners’ perceptions of love using a Quasi-Signal Detection approach (Reis et al., 2014) (see OSM for key conceptual distinctions from the current research). The sample size was determined prior to data collection based on contemporary standards for large samples.

9. Given the marginal effect and prior work documenting gender differences in studies of partner judgments for interaction traits (e.g., Fletcher & Kerr, 2010), we tested for gender moderations across studies and report these estimates in the OSM. Notably, gender did not moderate the main effects of directional bias, tracking accuracy, or assumed similarity in either study.

10. A sensitivity analysis estimated the sample size necessary to conduct DRSA in our model following guidelines suggesting two to three times as many participants needed to detect linear effects in a standard APIM (Schönbrodt et al., 2018). Using parameters obtained from Study 1, simulation results indicated at least 59 dyads for 80% power to detect medium size (*r* = .25) effects; the 175 dyads in Study 2 constitute almost three times this sample size (see OSM).
