# Supplementary material for: Half Empty and Half Full? Biased Perceptions of Compassionate Love and Effects of Dyadic Complementarity
Source: Pers Soc Psychol Bull. 2023 May 26;50(10):1423–37. doi: 10.1177/01461672231171986 (PMC11367802; doi:10.1177/01461672231171986)
Supplement: sj-docx-1-psp-10.1177_01461672231171986 – Supplemental material for Half Empty and Half Full? Biased Perceptions of Compassionate Love and Effects of Dyadic Complementarity [file sj-docx-1-psp-10.1177_01461672231171986.docx]

**Online Supplemental Materials**

**Additional Notes on Prior Examinations of Bias/Accuracy for Interaction Attributes**

Among the few empirical studies specifically examining judgments of purported interaction attributes, findings have not painted a clear picture as to whether both partners are equally benefited from such biased perceptions. Muise et al. (2016) found that a biased tendency (in men, specifically) to perceive less sexual desire than their romantic partner reported was positively associated with their partner’s relationship satisfaction. Similarly, LaBuda and Gere (2021) identified an underestimation effect for partner’s approach sacrifice motives, which predicted greater closeness felt by one’s partner but less closeness felt by oneself (i.e., the perceiver). In work by Dobson and colleagues (2021), people’s perceptions of sexual rejection by their partner—a negative interactional quality—were characterized by overestimation, which was associated with lower satisfaction for perceivers. Furthermore, a few studies have yielded somewhat inconsistent results for other interactional quality-type attributes. For example, no significant negative bias was found for partner’s communal behavior (Pusch et al., 2020) or relationship authenticity (Wickham & Bond, 2020); however, the effects were in the expected direction. Bar-Kalifa et al., 2016, found positive bias (i.e., overestimation) for judgments of partner support behaviors, though the reasons for this are unclear.

**Study 1 Sensitivity Analysis for DRSA**

A sensitivity analysis was conducted to estimate the sample size necessary to conduct DRSA in our specific model by following general recommendations (Schönbrodt et al., 2018). Conducting power analysis in DRSA is not trivial as it requires fully specifying all (co)variance and path coefficients of an assumed population model (including estimation of quadratic effects), most of which were difficult to ascertain. Some of these parameters, however, were determined from Study 1 results (i.e., actor-partner covariances between the predictor and outcome variables). We used the APIM power application (Ackerman et al., 2016) in combination with the general guidelines recommending two to three times as many participants for higher-order terms as needed to detect linear effects of the predictors (Aiken & West, 1991; Schönbrodt et al., 2018). DRSA guidelines suggest a minimum sample size of two to three times as many participants needed to detect linear effects in a standard APIM (Schönbrodt et al., 2018). Results indicated at least 59 dyads for 80% power to detect medium size (*r* = .25) linear effects, suggesting 118 to 177 dyads for DRSA. Thus, the 56 dyads in Study 1 was insufficiently powered for this method. Study 2 consisted of 175 dyads, roughly three times this sample size, thus meeting criteria for adequate power for DRSA.

**Study 1 Sensitivity Analysis Additional Details**

We conducted a sensitivity analysis to determine how much power we had in our design (i.e., 56 couples in a 14-day diary) to detect our key effects of interest: the main T&B effects and main effects of next-day actor and partner satisfaction. The latter two represent whether directional bias is moderated by next-day actor and partner satisfaction in our satisfaction T&B models. Of particular interest was probing the partner satisfaction effect (non-significant in Study 1) to determine the sample size necessary to detect the effect with 80% power in Study 2. The PowerLAPIM app was used (Lafit et al., 2022), entering estimates from our satisfaction model to assess sensitivity for a model with a continuous time-varying moderator for indistinguishable analysis (i.e., Model 6 in the app). The following estimates were entered into the app from the satisfaction model run in Study 1 using 1000 Monte Carlo replications at alpha = .05: Number of dyads: 56, Number of time points: 14, Fixed intercept (i.e., directional bias): -.18, Fixed actor effect (i.e., assumed similarity): .80, Fixed partner effect (i.e., tracking accuracy): .14, Effect of time-varying continuous variable C (i.e., partner satisfaction effect): -.03, Moderation of C on actor effect: -.003, Moderation of C on partner effect: -.04, SD of Level 1 errors for partners A and B: .59, Level 1 errors correlation: -.09, SD of random intercept: .34, Mean of time-varying variable X for partner A and B: 0, SD of X for partner A: 1.11, SD of X for partner B: .69, Correlation between X for partners: .64, Mean of C: 0, SD of C: 1.32.

Results showed 72% power to detect the minimum effect size observed among the key partner effects in the T&B model. It was determined that 67 participants dyads would be required to attain 80% power in a similar study design. The sample in Study 2 well exceeded this threshold, providing adequate power to investigate the main effect of partner satisfaction more thoroughly. Sensitivity analyses determined there was at least 80% power to detect all other key effects of interest in the T&B models (i.e., the effect of directional bias, assumed similarity, tracking accuracy, and the effects of directional bias on actor satisfaction).

**Comparisons with Grand-Mean Centered T&B Analyses**

Person-mean centered results reported in the article were largely consistent with results based on grand-mean centering, which we report here. In these models, perceivers’ ratings of their partner are compared with their partner’s actual ratings as well as their own ratings. All variables (i.e., perceivers’ judgments of their partner’s daily love behaviors, partners’ reported love behaviors, and perceivers’ reported love behaviors) were centered on the grand mean of enacted love behaviors in the sample.

In Studies 1 and 2, we again found significant underestimation of partner love behaviors in relationships, as well as significant effects of tracking accuracy and assumed similarity, consistent with hypotheses (see Tables S1-S3). In Study 1, greater underestimation was related to significantly lower daily satisfaction for actors (*b* = .06, *p* = .005) but higher daily satisfaction for partners, although this latter effect was not significant (*b* = -.032, *p* = .122).

**Table S1**

*T&B effects (grand-mean centered) in Studies 1 & 2*

|  | ***b*** | ***SE*** | ***t*** | ***p*** |
| --- | --- | --- | --- | --- |
| **Study 1** |  |  |  |  |
| Directional Bias | -.171 | .032 | -5.377 | <.001 |
| Tracking Accuracy | .872 | .021 | 42.250 | <.001 |
| Assumed Similarity | .175 | .020 | 8.674 | <.001 |
| **Study 2** |  |  |  |  |
| Directional Bias | -.252 | .061 | -4.122 | <.001 |
| Tracking Accuracy | .552 | .013 | 41.668 | <.001 |
| Assumed Similarity | .292 | .013 | 22.052 | <.001 |

**Table S2**

*Study 1 T&B associations with actor-partner satisfaction (grand-mean centered)*

|  | ***b*** | ***SE*** | ***t*** | ***p*** |
| --- | --- | --- | --- | --- |
| **Effects of T&B Components** |  |  |  |  |
| Directional Bias | -.181 | .034 | -5.308 | <.001 |
| Actor Satisfaction | .060 | .021 | 2.825 | .005 |
| Partner Satisfaction | -.032 | .020 | -1.550 | .122 |
| Tracking Accuracy | .111 | .025 | 4.458 | <.001 |
| Actor Satisfaction | .011 | .012 | .860 | .390 |
| Partner Satisfaction | -.020 | .012 | -1.666 | .096 |
| Assumed Similarity | .755 | .026 | 28.994 | <.001 |
| Actor Satisfaction | .020 | .013 | 1.561 | .119 |
| Partner Satisfaction | -.007 | .013 | -.588 | .557 |

**Table S3**

*Study 2 T&B associations with actor-partner satisfaction (grand-mean centered)*

|  | ***b*** | ***SE*** | ***t*** | ***p*** |
| --- | --- | --- | --- | --- |
| **Effects of T&B Components** |  |  |  |  |
| Directional Bias | -.292 | .061 | -4.812 | <.001 |
| Actor Satisfaction | .099 | .040 | 2.443 | .015 |
| Partner Satisfaction | -.075 | .040 | -1.855 | .064 |
| Tracking Accuracy | .248 | .015 | 17.115 | <.001 |
| Actor Satisfaction | .008 | .011 | .727 | .467 |
| Partner Satisfaction | .006 | .011 | .540 | .589 |
| Assumed Similarity | .432 | .015 | 29.804 | <.001 |
| Actor Satisfaction | .008 | .011 | .732 | .464 |
| Partner Satisfaction | .005 | .011 | .432 | .666 |

**Lagged Models Predicting Current-Day Satisfaction**

Estimates below are from lagged models predicting today’s (i.e., current-day) actor-partner satisfaction controlling for the previous day’s satisfaction. Study 1 results were highly consistent, with the sole differences being that the tracking accuracy effect on partner satisfaction was no longer significant, while the assumed similarity effect on actor satisfaction was significant. Likewise, Study 2 results were highly consistent, with the sole differences being that the assumed similarity effects on actor and partner satisfaction were significant. See Table S4 below.

**Table S4**

Lagged T&B models predicting current-day satisfaction (controlling for previous day satisfaction)

|  | ***b*** | ***SE*** | ***t*** | ***p*** |
| --- | --- | --- | --- | --- |
| **Study 1** |  |  |  |  |
| Directional Bias | -.174 | .037 | -4.738 | <.001 |
| Actor Satisfaction | .225 | .021 | 10.749 | <.001 |
| Partner Satisfaction | .022 | .021 | 1.093 | .275 |
| Tracking Accuracy | .076 | .028 | 2.678 | .008 |
| Actor Satisfaction | -.001 | .017 | -.077 | .939 |
| Partner Satisfaction | -.022 | .019 | -1.193 | .233 |
| Assumed Similarity | .811 | .025 | 32.323 | <.001 |
| Actor Satisfaction | .059 | .011 | 5.141 | <.001 |
| Partner Satisfaction | -.014 | .011 | -1.349 | .178 |
| **Study 2** |  |  |  |  |
| Directional Bias | -.293 | .057 | -5.150 | <.001 |
| Actor Satisfaction | 1.030 | .044 | 23.353 | <.001 |
| Partner Satisfaction | -.229 | .043 | -5.288 | <.001 |
| Tracking Accuracy | .223 | .016 | 14.356 | <.001 |
| Actor Satisfaction | .002 | .014 | .108 | .914 |
| Partner Satisfaction | -.005 | .013 | -.406 | .685 |
| Assumed Similarity | .420 | .015 | 28.514 | <.001 |
| Actor Satisfaction | .039 | .010 | 3.998 | <.001 |
| Partner Satisfaction | .022 | .010 | 2.176 | .030 |

**Distinctions from Reis et al. (2014)**

In Reis et al. (2014), the authors used a Quasi-Signal Detection (QSD) approach to examine the consequences of partners accurately detecting (‘hits’), failing to detect (‘miss’), or falsely detecting (‘false alarm’) partner’s specific love behaviors. Relevant to the current research, the authors found that enacting and perceiving CL acts predicted greater actor and partner satisfaction, and that men reported enacting more CL behaviors than women reported perceiving across the entire sample. Using the QSD approach, they found that actor’s and partners’ hits, misses, and false alarms all predicted greater satisfaction (with only one effect not reaching significance). Subsequent analyses comparing the strength of actor and partner effects suggested perceivers benefitted more from having their CL behaviors mutually recognized (i.e., accurately perceived) or falsely detected (i.e., overestimated) by their partner, but when their CL behaviors were missed (i.e., underestimation), they benefitted less relative to their partner. Although Reis and colleagues’ (2014) findings are informative to the current research topic, the Truth and Bias Model used here carries a number of strengths compared to the QSD approach with respect to our specific research aims. First, it can better capture the different components of accuracy and bias for perceptions of partner love, such as whether partners tend to be systematically biased in their judgments of partner love, as well as the degree to which partners can accurately track overall levels of partner love. In other words, the T&B method can systematically parse out these effects from one another unlike in a QSD approach, which is more suitable for examining outcomes associated with distinct events rather than general patterns of perception. Second, in QSD, the effects of hits, misses, and false alarms are interpreted relative to instances of when a CL behavior was not enacted (i.e., a ‘correct rejection’); for example, this study found that having one’s CL behavior ‘missed’ was found to predict higher—as opposed to lower—satisfaction (since a CL behavior was enacted at all). Interpreting the effects of the different categories in relation to one another is more challenging with this approach, making it less informative for addressing our second research question. Further, and perhaps most significantly, a T&B approach can incorporate the effect of projection, which Reis and colleagues (2014) identified as a future direction for research.

**Estimates from Gender Moderation Tests of Main T&B Models**

As displayed below in Table S5, gender did not significantly moderate the main T&B effects in both studies (*p*s > .05), thus follow-up analyses were limited. Study 2 showed gender differences only for the variance estimates between men and women, but as mentioned, gender did not significantly moderate any of the main effects.

We additionally examined mean level differences in men’s and women’s daily expressions of compassionate love across their diaries through independent samples *t*-tests. Results indicated that in Study 1, there was no significant difference between the average levels of daily compassionate love reported by men (*M* = 5.84, *SD* = .99) and women (*M* = 5.54, *SD* = .96), *t*(110) = 1.64, *p* = .104, CI_95%_ [-.06, 0.67]. In Study 2, men engaged in significantly more daily compassionate love (*M* = 5.95, *SD* = 2.04) than women (*M* = 6.49, *SD* = 2.23), *t*(348) = 2.36, *p* = .019, CI_95%_ [-.09, 0.99]; however, this effect was small (*d* = .25).

**Table S5.**

T&B Model Estimates Testing for Gender Interactions

|  | ***b*** | ***SE*** | ***t*** | ***p*** |
| --- | --- | --- | --- | --- |
| **Study 1 Gender Interactions** |  |  |  |  |
| Directional Bias | .003 | .037 | .087 | .930 |
| Tracking Accuracy | .041 | .024 | 1.697 | .090 |
| Assumed Similarity | -.007 | .021 | -.336 | .737 |
| **Study 2 Gender Interactions** |  |  |  |  |
| Directional Bias | -.098 | .093 | -1.049 | .294 |
| Tracking Accuracy | .017 | .014 | 1.226 | .220 |
| Assumed Similarity | .023 | .012 | 1.837 | .066 |

*Note.* See supplemental analyses output file to view all estimates in the model.

**Auxiliary Analyses of daily satisfaction models with between-partner correlation fixed to zero**

Results showed a within-couple correlation between partners’ directional biases. To assess whether this correlation accounts for the daily satisfaction effects (i.e., actor underestimation is associated with partner overestimation, which in turn predicts greater partner satisfaction), we re-ran our satisfaction models fixing the correlation between partner random effects to zero (i.e., the within-dyad random intercept correlation). All of the reported results held, suggesting these effects are not driven by partner complementarity in directional bias:

**Table S4**

*Fixed within-couple correlation for Study 1 T&B daily satisfaction* *model*

|  | ***b*** | ***SE*** | ***t*** | ***p*** |
| --- | --- | --- | --- | --- |
| **Effects of T&B Components** |  |  |  |  |
| Directional Bias | -.178 | .037 | -4.741 | .000 |
| Actor Satisfaction | .041 | .020 | 2.047 | .041 |
| Partner Satisfaction | -.029 | .019 | -1.518 | .129 |
| Tracking Accuracy | .135 | .028 | 4.861 | .000 |
| Actor Satisfaction | .023 | .018 | 1.261 | .208 |
| Partner Satisfaction | -.041 | .019 | -2.127 | .034 |
| Assumed Similarity | .799 | .025 | 31.881 | .000 |
| Actor Satisfaction | .014 | .012 | 1.171 | .242 |
| Partner Satisfaction | -.003 | .011 | -.296 | .767 |

**Table S5**

*Fixed within-couple correlation for Study 2 T&B daily satisfaction* *model*

|  | ***b*** | ***SE*** | ***t*** | ***p*** |
| --- | --- | --- | --- | --- |
| **Effects of T&B Components** |  |  |  |  |
| Directional Bias | -.279 | .076 | -3.657 | .000 |
| Actor Satisfaction | .104 | .041 | 2.562 | .010 |
| Partner Satisfaction | -.123 | .040 | -3.031 | .003 |
| Tracking Accuracy | .235 | .016 | 15.126 | .000 |
| Actor Satisfaction | -.001 | .014 | -.063 | .950 |
| Partner Satisfaction | .022 | .014 | 1.609 | .108 |
| Assumed Similarity | .453 | .014 | 31.644 | .000 |
| Actor Satisfaction | .004 | .010 | .372 | .710 |
| Partner Satisfaction | .009 | .010 | .862 | .389 |

**Conceptualization of Dyadic Complementarity in DRSA**

As noted in the manuscript, complementarity effects have been conceptualized in different ways in the literature. The current approach emphasizes the qualitative *pattern* of complementarity (mismatching across all levels whereby Y = -X) rather than the degree of complementarity (more versus less extreme levels of mismatching). We are aware of two other studies that have conceptualized a complementary hypothesis within a RSA framework (Cheung, Chiu, & Choi, 2022; Xie, Shi, & Ma, 2017). The authors of these papers principally conceptualize complementarity according to a curvilinear effect along the line of incongruence (LOIC), thus interpreting a positive a4 parameter as support for their complementarity hypothesis. Here, we acknowledge that this interpretation can reflect a complementarity effect, but contend that a significant a4 *qualifies*a pattern of complementarity. Insofar as a congruence effect (i.e., similarity hypothesis) in RSA is principally operationalized by Humberg et al. (2019) as the ridge of the response surface lying along the line of congruence (LOC), we understand the logical inverse of this to consist of the ridge lying along the LOIC, designated by the a2 parameter more so than the a4. Thus, our conceptualization of a complementarity effect considers dissimilarity as an overall pattern, such that perfect mismatching (i.e., Y = -X) predicts an outcome across all levels regardless of degree. In contrast, it may be the case that complementarity among predictor variables is expected to be ‘level-dependent’, such that more versus less extreme degrees of mismatching predict higher levels of an outcome. Although we consider both cases to reflect complementarity effects, we contend that whether the outcome depends on the degree of dissimilarity—as in Cheung et al. (2022) and Xie et al. (2017)—is not essential to defining complementarity. In other words, whether ‘opposites’ are better does not necessarily entail that more extreme opposites are better.

**Figure S1. Study 2 Directional Bias Scores in Sample**

**
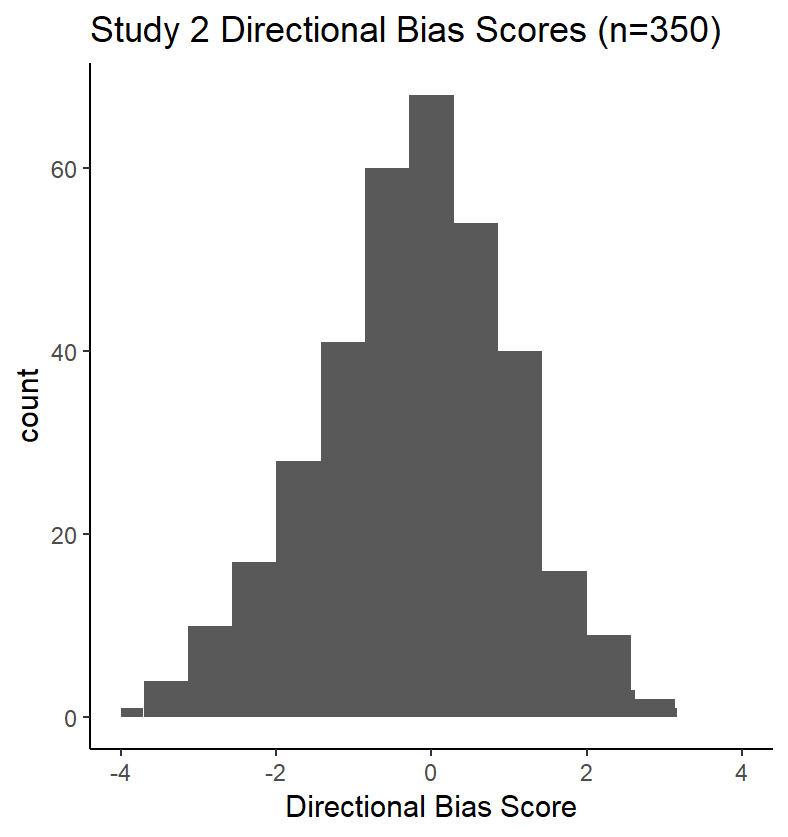
**

**Gender-Constrained Model Tests for Study 2 DRSA**

The resulting DRSA model in Study 2 consisted of imposing constraints to estimate equal intercepts, residual variances, predictor means/variances, and actor-partner effects between couple members according to their gender (Schönbrodt et al., 2018). Formally, the full DRSA model is defined by two polynomial regressions, where the same pair of predictor variables (i.e., men’s and women’s directional bias), their interaction, and their squared terms predict men’s and women’s outcome variables, with error terms correlated to account for the non-independence between dyad members (see Equation 3 from Schönbrodt et al., 2018 below):

*Z_f_ = b_0f_ + b_1f_X + b_2f_Y + b_3f_X^2^ + b_4f_XY + b_5f_Y^2^ + e_f_*

*Z_m_ = b_0m_ + b_1m_X + b_2m_Y + b_3m_X2 + b4_m_XY + b_5m_Y^2^ + e_m_*

*e_f_∼∼e_m_*

If men’s and women’s effects are not significantly different from one other, the respective paths can be set equal, which is advantageous for increasing the sample size and the power to detect smaller effects, as well as allowing for more parsimonious interpretations of the data. Following recommended guidelines (Schönbrodt et al., 2018), we computed the full DRSA model in a SEM framework using the *lavaan* package in R. For the gender-constrained model, which assumes that the response surface is the same for both genders, we fixed the actor effects, partner effects, and interaction effect path coefficients to be equal for both genders (i.e., *b_1f =_ b_2m_ , b_3f =_ b_5m_ , b_2f =_ b_1m_ , b_5f =_ b_3m_ , b_4f =_ b_4m_*) to run model comparisons and obtain support for an indistinguishable model. The final reported indistinguishable model set the predictor variables (i.e., men’s and women’s directional bias) to have equal means and variances, and the outcome variables (i.e., men’s and women’s relationship satisfaction) to have equal residual variances and intercepts (Schönbrodt et al., 2018).

**Additional Details (Study 1 and Study 2)**

The current research analyzes couples data which were not collected specifically for the purposes of the current research. Although data for Study 1 were collected after Study 2 (and different measures of compassionate love were used between studies), the present research questions and analytic approach were developed after data collection had finished for both studies. We have presented Study 1 and Study 2, not in the chronological order in which the data were collected, but according to narrative fluency and relative complexity in their ability to address the research questions of interest (e.g., DRSA is conducted in Study 2 alone).
